# Computational characterization of halogen vapor attachment, diffusion and desorption processes in zeolitic imidazolate framework-8

**DOI:** 10.1038/s41598-020-59871-x

**Published:** 2020-02-20

**Authors:** Dejie Li, Ying Han, Deqiang Li, Qi Kang, Dazhong Shen

**Affiliations:** 1 College of Chemistry, Chemical Engineering and Materials Science, Collaborative Innovation Center of Functionalized Probes for Chemical Imaging in Universities of Shandong, Key Laboratory of Molecular and Nano Probes, Ministry of Education, Shandong Provincial Key Laboratory of Clean Production of Fine Chemicals, Shandong Normal University, Jinan, 250014 P. R. China; 2 National Engineering Research Center for Colloidal Materials and School of Chemistry and Chemical Engineering, Shandong University, Jinan, 250100 China

**Keywords:** Computational chemistry, Method development

## Abstract

Computational simulation methods are used for characterizing the detailed attachment, diffusion and desorption of halogen vapor molecules in zeolitic imidazolate framework-8 (ZIF-8). The attachment energies of Cl_2_, Br_2_ and I_2_ are −55.2, −48.5 and −43.0 kJ mol^−1^, respectively. The framework of ZIF-8 is disrupted by Cl_2_, which bonds with Zn either on the surface or by freely diffusing into the cage. A framework deformation on the surface of ZIF-8 can be caused by the attachment of Br_2_, but only reorientation of the 2-methylimidazolate linkers (mIms) for I_2_. In diffusion, the halogen molecules have a tendency to vertically permeate the apertures of cages followed with swing effect implemented by the mIms. Larger rotation angles of mIms are caused by Br_2_ because of its stronger interaction with mIms than I_2_. A maximum of 7 Br_2_ or 5 I_2_ molecules can be accommodated in one cage. Br_2_ are clinging to the mIms and I_2_ are arranged as crystal layout in the cages, therefore in desorption processes molecules attached to the surface and free inside are desorbed while some remained. These results are beneficial for better understanding the adsorption and desorption processes of halogen vapors in the porous materials.

## Introduction

The research subjects in the preparation, characterization and study, related to metal–organic frameworks (MOFs), have been explosive growth^1^. MOFs possess unique property among the various microporous and mesoporous materials, creating interest for an unprecedented range of applications^2^. As a new class of crystalline nanoporous materials, MOFs have been found extensively applied in the areas such as catalysis^3^, biomedicine^4^, separation^5^, gas storage^6^, chemical sensing^7^, air purification^8^, imaging agents^9^, and so on.

One of the outstanding characteristics of MOFs are their highly diverse conformation, ideal porosity, and good chemical properties^10^, making them suitable for gas adsorption or separation and being a substitute for traditional adsorbents^11^. For example, Khan *et al*. used multinuclear solid-state NMR to study the adsorption of biologically important signaling molecule NO on two MOFs, indicating both physisorption and chemisorption of NO adsorbed on the open metal site^12^. Niu *et al*. successfully synthesized hierarchical UiO-66 and its amino-analog UiO-66-NH_2_ nanocrystals can enhance the adsorption capacity by introducing water into the conventional synthesis without the need for a chelating agent or surfactant^13^.

To better understand the mechanism governing the gas molecules migration in adsorption processes or establish an investigation approach to promote the adsorption properties of the absorbents, detailed information on the gas adsorption and diffusion processes is crucial^14^. Therefore, nowadays more and more attention has been paid to the role of computational simulation in the structure design and performance evaluation of MOFs^15^. Kitao *et al*. provided new and versatile MOFs by simulation that exhibit peculiar properties hard to realize with the individual components^16^. Farha *et al*. used computational modeling to design and characterize predictively MOFs with high surface areas^17^.

Zeolitic imidazolate frameworks (ZIFs) are a sub-class of MOFs. Zeolitic imidazolate frameworks-8 (ZIF-8) is a compelling example because of its high thermal and chemical stability^18^. Bonding between 2-methylimidazolate linkers (mIms) and Zn^2+^ constituting the node is comparatively strong and the physical shielding of Zn^2+^ by four tetrahedrally coordinated mIms is effective^19^. The apertures in the framework lack polar groups and exclude liquid water effectively^20^. Therefore, ZIF-8 can bear prolonged soaking in 8 M NaOH solution at 100 °C^21^. It is suitable for gas separation/adsorption at temperatures below 300 °C in the presence of air/water and under inert gas over 400 °C^22^. Many researchers are attracted by the stability to study the application of ZIF-8 as a gas adsorbent^23^.

Halogens are playing important roles in many aspects of industrial processes. Cl_2_ is used as disinfectant in water supply system and is an ingredient of plastic products^24^. Br_2_ and I_2_ are key elements in medicine, photography and dyes^25^. However, the safe handling, storage and transportation of halogens are great challenges because of their toxicity, corrosiveness and volatility. It is necessary to study the safe use or disposal of halogen gases such as adsorption. In the gas adsorption applications, ZIF-8 is usually chosen as the adsorbent^26^. The dynamic adsorption and desorption processes of Br_2_ and I_2_ on ZIF-8 film have also been monitored by electrodeless quartz crystal microbalance (EL-QCM) technique in our previous experiments^27,28^.

In this work, for the purpose of supplying more detailed information and establishing a theoretical model to promote the adsorption properties of the adsorbents through modification, computational simulation methods are used to investigate the Br_2_ and I_2_ adsorption/desorption processes in ZIF-8 on thermodynamics and dynamics. Additionally, the interaction of Cl_2_ and ZIF-8, which is scarcely discussed to our knowledge, is also included. Energy variations, charge distributions and structure parameters are explained and some of the results are confirmed by experiments. It is hopeful that the detailed characterization, could serve as an important stepping stone for understanding the adsorption/desorption processes during uptake action, illuminating the way of theoretical prediction or design on the porous material performances.

## Methods

From the microscopic viewpoint, ZIF-8 crystal is characterized by the presence of square and hexagonal windows around the main pore. Calculation research performs on periodic system, which is uncharged compared to the fragments that Zn atoms at the vertices of square or hexagonal windows are fixed^29^. As previous research done^30^, hexagonal window portion of the lattice was adopted in this work (Fig. S1, Supplementary Information). Previous research investigations^31,32^, employed the density functional theory (DFT) method with B3LYP/lanl2dz basis set to obtain a much better energy description of the intermolecular interaction between ZIF-8 and vapors. The results are comparable with that calculated by Moller-Plesset correlation energy correction truncated at second-order and higher-level ab initio methods, but at a much lower computational effort^33^. Thus in this work, the simulation was performed by using the same basis set and all the results were zero-point corrected (*ZPC*) energies.

Stationary points calculated had been characterized as minima state or activation state by analyzing the vibrational normal mode^32^. That point could be classified as minima if no imaginary frequency was obtained while an activation state if only one imaginary frequency was observed^33^. Atomic charges in the aperture structures were obtained by the natural population analysis (NPA) of Weinhold^34^. The attachment energy (*E*_T_) of halogen molecule at each adsorption site was calculated from the energy difference between the stable structure with adsorbed molecule [*E* (ZIF-8 + gas)] and the stable empty structure [*E* (ZIF-8)] corrected by the energy of the free molecule for the same volume [*E* (gas)]. That is, *E*_T_ = *E* (ZIF-8 + gas) − *E* (ZIF-8) − *E* (gas). All the simulated work was performed with GAUSSIAN 09 suite of package^35^.

Additionally, to gain more insights into the diffusion processes of Cl_2_, Br_2_, and I_2_ in ZIF-8, the molecular dynamics (MD) simulation was also performed. The crystal structure of ZIF-8 was obtained from the previously published literature^36^. Due to large computational cost, geometric optimization was performed by Forcite module^37^ based on the Universal Force Field (UFF)^38^ in Materials Studio software. Equilibrium MD simulation was used to calculate self-diffusivities in ZIF-8 and it was also implemented in the Forcite module^39^. Independent simulation was performed for each process with NVT ensemble. The total simulation time was 2 ns with a time step of 1 fs. 1 ns was used for equilibrium, another 1 ns for statistics.

## Results and Discussion

To describe more accurately, the whole processes can be divided into three processes: attachment (halogen molecule attaches to the aperture), diffusion (halogen passes through the aperture) and desorption (the opposite process of attachment and diffusion).

### Selection of the aperture structures

Due to the size of the aperture windows (diameter, 3.40 Å) which connect the large pores (diameter, 11.60 Å), ZIF-8 is recognized as a molecular sieve^40,41^. In gas adsorption process, the high diffusivity implies the swing effect of mIms during the gas uptake experiments^42^. This structure variation is fundamentally important because it promotes the diffusivity of gases through the porous networks^43^. Based on the flexibility stated above, 8 different aperture structures are designed to select the appropriate simulation model (Fig. S2), of which the methyl in the mIms (Me-m) point to different directions.

In the aperture (Fig. S2), structure of all Me-m oriented to Z-axis is named structure A, abbreviated S-A; structure B (S-B), Me-m 1 oriented to Z-axis; structure C (S-C), Me-m 1, 2 oriented to Z-axis; structure D (S-D), Me-m 1, 3 oriented to Z-axis; structure E (S-E), Me-m 1, 4 oriented to Z-axis; structure F (S-F), Me-m 1, 3, 5 oriented to Z-axis; structure G (S-G), all Me-m oriented to the center; structure H (S-H), all Me-m oriented against to the center. Other Me-m not mentioned oriented against the Z-axis. Structures simulated are originally composed of mIms groups, to show clearly, in figures the branched mIms are removed.

**Figure 1:**
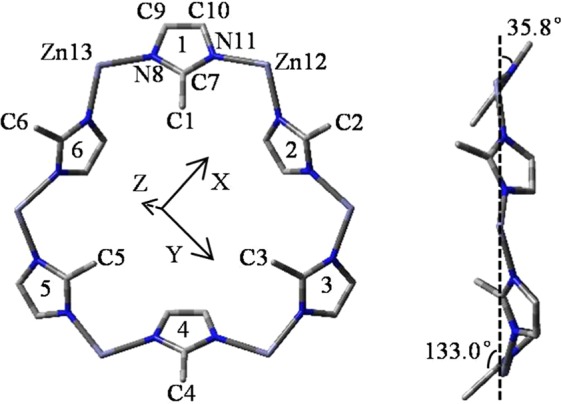
Vertical and side views about the most stable structure of the relevant hexagonal window aperture of ZIF-8. Zn, C and N atoms are shown in pink, gray and blue colors, respectively. H are omitted for clarity in the figure. All the six Zn form a surface which represented by X, Y-axis and the mIms are numbered from 1 to 6. Bond distances are in Å.

*ZPC* energies of S-A to S-H based on S-A (0 kJ mol^−1^) are listed in Table 1. The energies are 0 (S-A), 11.8 (S-B), 11.1 (S-C), 8.1 (S-D), 4.6 (S-E), −0.5 (S-F), 10.3 (S-G) and 20.2 (S-H), respectively. Therefore, S-F is the most stable structure. The vertical and side views of S-F is shown in Fig. 1. All the Zn form a plane represented by X and Y-axis. The mIms are numbered from 1 to 6 and mIm 1, 3, 5 follow by a 35.8° rotation, in good agreement with the previous research reported^44^. Detailed bond lengths (Å) and charge distributions (a.u.) are compiled in Tables S1 and S2.

**Table 1:** Zero-point corrected *(ZPC)* energies (kJ mol^−1^) of 8 aperture structures optimized.

Structures	S-A	S-B	S-C	S-D	S-E	S-F	S-G	S-H
Energy	0	11.8	11.1	8.1	4.6	−0.5	10.3	20.2

### Attachment and diffusion processes of Cl_2_

**Figure 2:**
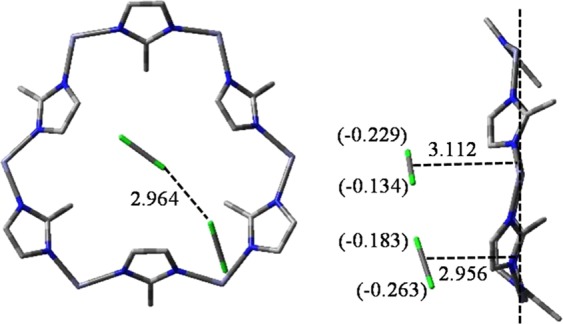
Vertical and side views about the stable structure of two Cl_2_ molecules attachment on the aperture. Bond distances are in Å and the charges are listed in parentheses.

**Figure 3:**
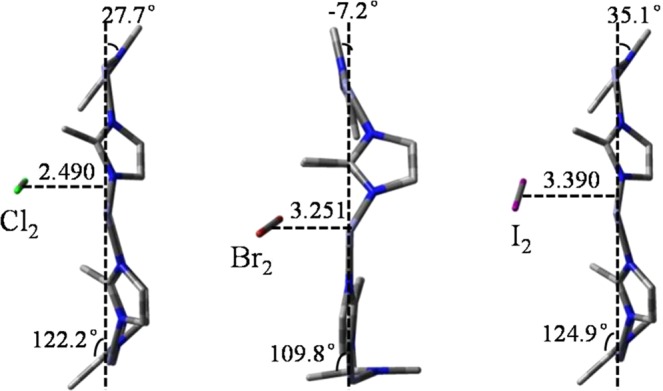
Side views about the stable structures of halogens attachment on the aperture. Bond distances are in Å.

Since the diatomic bond length of Cl_2_ is short (2.224 Å), optimized result reveals that at the same time, several Cl_2_ molecules gather together on the top of the aperture (Fig. S3(a)). As the halogen gas getting closer and closer to the aperture, charges on each Cl are changing to negative. At some point in time at least up to two Cl_2_ molecules can be accommodated by the aperture, as shown in Fig. 2. Charge distributions on each Cl are −0.229 and −0.134, −0.183 and −0.263, respectively. Distance between two molecules is 2.964 Å. The attraction established between Cl_2_ and the aperture is 18.9 kJ mol^−1^ in average. Therefore, Cl_2_ are still on the way of approaching to the aperture at that time. As Cl_2_ getting closer to the aperture, a site on top of the aperture center where only one Cl_2_ molecule located can be identified. The stable structure is shown in Fig. 3. The diatomic bond of Cl_2_ is parallel to the plane of the aperture and the distance between Cl_2_ and the aperture is 2.490 Å. In general, the attachment energies of H_2_, CO_2_ and CH_4_ are −8.6, −20.0 and −20.9 kJ mol^−1^, respectively, and charges of the aperture change a little^28,29,45^. However, in this structure, the polarizability of Cl_2_ is increased and the charge distributions change to −0.240 and −0.257, respectively. The averaged charge on protons surrounding increases from 0.027 to 0.042. Charge variation in this structure is greater than that of other gases stated above. The attachment process of Cl_2_ releases more energy and *E*_T_ is −55.2 kJ mol^−1^. It is indicated that the attachment of Cl_2_ vapor is a spontaneous process.

**Figure 4:**
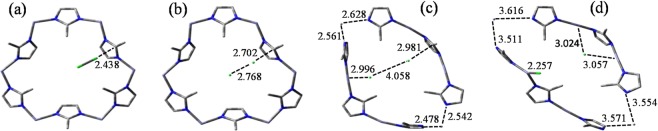
The disruption profile of aperture in the diffusion process of Cl_2_. (**a**–**d**) are four typical structure change nodes. Bond distances are in Å.

In the most stable aperture, charge distribution on C7 is 0.456. At the beginning of diffusion process, MD simulation reveals that there is an interaction between Cl_2_ and C7 (Fig. S4). Related structures are further calculated by using the more precise DFT method. As shown in Fig. 4(a), Cl_2_ is close to the plane of the aperture in the initial. Then it is attracted to interact with C7 and the distance between them is 2.702 Å, as shown in Fig. 4(b). At that time, charge distributions on two Cl change to −0.482 and −0.449, respectively. Two Cl depart from each other and the distance is 2.768 Å. This distance keeps getting longer and longer. In Fig. 4(c), the positive part on the other side of the aperture, especially Zn which has a charge of 0.870, is attracted by the free Cl and the aperture is distorted. One Cl still interacts with C7 and the other interacts with Zn on the other side. Correspondingly, charge redistribution of the aperture is occurred. For example, charge distribution on N8 changes from −0.811 to 0.481. Bond lengths of N–Zn are dramatically elongated and bond strengths are greatly weakened. Finally, with structure adjustment, some bond lengths of N–Zn are extended more than 3.511 Å and the aperture is disrupted, as shown in Fig. 4(d).

**Figure 5:**
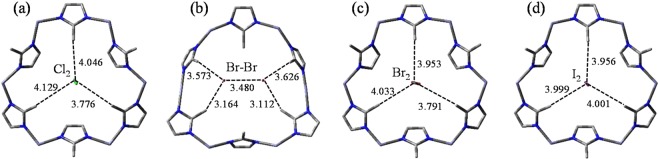
Vertical views about the structures of halogens diffusion in the aperture. Bond distances are in Å. (**a**) Cl_2_ diffusion in the aperture, (**b**,**c**) different states of Br_2_ diffusion in the aperture, (**d**) I_2_ diffusion in the aperture.

It is particularly noteworthy that there exists another type of interaction that can be regarded as an activation state in diffusion process of Cl_2_. In Fig. 5(a), structure with an imaginary frequency of 475 *i* cm^−1^ is accompanied by the rotation of Cl_2_. The aperture plane separates Cl_2_ molecule into two parts: one Cl is pointing inside and the other pointing outside. Charge distribution on each Cl are −0.230 and −0.229, therefore, slight rotations of mIms are caused by the steric electric effect. However, distances between Cl_2_ and mIms 1, 3, 5 are 4.046, 3.776 and 4.129 Å, respectively, that are too long to cause an interaction between Cl_2_ and the aperture. There is a possibility that Cl_2_ can get through the apertures vertically and get inside the cages to bond with Zn. The disruption of ZIF-8 is confirmed by X-ray diffraction (XRD) and Fourier transform infrared spectroscopy (FTIR) investigations^46^. The spectrum in previous work reveals that structures of mIms are dramatically changed and vibrational modes of Zn–N bonds disappeared after interaction with Cl_2_ gas. Therefore, Cl_2_ can bond with Zn either on the surface or by freely diffusing into the cage, resulting in the disruption of ZIF-8.

### Attachment, diffusion and desorption processes of Br_2_

As the gas getting closer to the aperture, MD simulation reveals that at some point in time three Br_2_ molecules can exist simultaneously close to the aperture. Charge distributions on each Br are −0.120 and −0.155, −0.107 and −0.178, −0.151 and −0.176, respectively (Fig. S3(b)). Br_2_ molecules are more polarized than that of Cl_2_. In the stable structure, because bond length of Br_2_ (2.510 Å) is longer than that of Cl_2_ (2.224 Å), space resistance does not allow two Br_2_ molecules to locate and only one on top of the aperture center is identified, as shown in Fig. 3. Charge distribution on Br close to protons is −0.399 and the other is −0.377, which induce all of the six mIms to rotate. Me-m 2, 4, 6 are rotated by 23.2° and correspondingly Me-m 1, 3, 5 are rotated by 43.0° to adapt to the new distribution of charges. The distance between Br_2_ and the aperture is 3.251 Å, which is 0.761 Å longer than that of Cl_2_. The interaction between Br_2_ and the aperture is reduced by charge redistribution and the long distance, although Br_2_ is strongly polarized. Correspondingly, the *E*_T_ of Br_2_ is −48.5 kJ mol^−1^, exothermic 6.7 kJ mol^−1^ less than that of Cl_2_. Therefore, in the experiment, the adsorption rate of Br_2_ is slower than that of Cl_2_ in the initial stage (data for Cl_2_ are not shown because of the disruption of ZIF-8).

It is particularly noteworthy that there exist two activation states in the diffusion process of Br_2_. One state is that Br_2_ keeps diffusing accompanied by the aperture deformation. In Fig. 5(b), distances between each Br and the closest mIm are 3.164 and 3.112 Å, respectively. The diatomic bond length of Br_2_ is stretched to 3.480 Å. Correspondingly, deformation of the aperture is caused by the steric electric effect. The other state (446 *i* cm^−1^) is that Br_2_ is perpendicular to the aperture plane, as shown in Fig. 5(c). Distances between Br_2_ and mIms 1, 3, 5 are 3.953, 3.791 and 4.033 Å, respectively. Charge distributions on each Br are −0.385 and −0.361, respectively, which cause a large steric electric effect to the adjacent protons. Therefore, an averaged 40.0° reorientation of the mIms occurs, indicating a swing effect of the aperture. When the molecule gets inside the cage, charge distribution on each Br are −0.019 and −0.011, respectively. The steric electric effect of Br_2_ is dispersed due to more interaction to the framework.

The adsorption processes of Br_2_ vapor on ZIF-8 film is monitored by EL-QCM. The schematic drawing of experimental setups employed for equivalent circuit parameters and the measurements are illustrated in Fig. S5. The shifts of the resonant frequency (Δ*f*) is recorded (Fig. S6) and the trend of adsorption/desorption can be directly reflected. As stated above, the *E*_T_ of Br_2_ is greatly exothermic, thus it can be found in the experiment that the frequency drops rapidly in the initial 10 min, corresponding to the rapid increase in adsorption. In diffusion process, simulation result reveals that there exist adverse factors including the deformation of aperture and reorientation of mIms. Therefore, the adsorption takes a long time (100 min) to approach the stable level.

**Figure 6:**
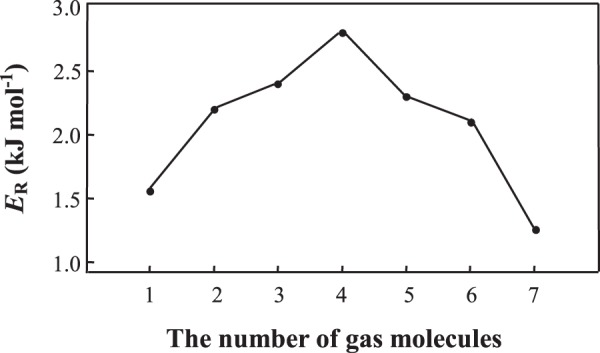
The released energies (*E*_R_) of Br_2_ placed in one cage with the molecule number of Br_2_ increasing from 0 to 7.

The released energies (*E*_R_) of Br_2_ molecules in one cage with the molecule number increasing from 0 to 7 are simulated. In Fig. 6, *E*_R_ is increased with the increase of gas molecules at the beginning. When 4 molecules are congregated inside, there is a repulsion to the new one. As the diffusion proceeding, distances between Br_2_ molecules are decreased (Fig. S7) and the repulsive forces increased (less than 2.0 kJ mol^−1^), thus *E*_R_ curve is descending. MD simulation shows that the framework of cage does not reveal any significant changes and most Br_2_ molecules are close to the cage walls. When the 8th molecule is diffused into the cage, there is an abnormal structure or energy interaction between Br_2_ and the cage. Additionally, the mass of Br_2_ inside the cages (g/g) can be obtained by Δ*f*^27^ in the experiment. Based on the molar mass of Br_2_ and cage unit, the molecular number ratio can be calculated. In the experiment, 7.3 Br_2_ can be obtained in each cage (0.87 g of Br_2_/g). Therefore, considering the results from simulation and experiment, it can be concluded that each cage can accommodate 7 Br_2_ molecules.

At the end of desorption process, approximately 21% of Br_2_ release is taken place and 79% left in the cages when the vacuum operation is carried out in the experiment (Fig. S6). Simulated result reveals that there is an interaction between Br_2_ and mIms. In general, it needs to take in the energy of 5.5 kJ mol^−1^ at least for Br_2_ inside the cage to escape from the grasp of mIms, indicating that mIms play an important role in Br_2_ adsorption. The simulated result is supported by Δ*f* measured in the experiment (Fig. S6). In the cage, the majority of Br_2_ interact with mIms and firmly attach to the cage walls; the minority are prone to move in the state of non-regular thermal motion. Therefore, in desorption process, the escaped vapor are the Br_2_ in the state of non-regular thermal motion in the cages and attached on the surface of ZIF-8 film before, while others stay inside.

### Attachment, diffusion and desorption processes of I_2_

In attachment process, one molecule is close to the aperture (the distance less than 4.0 Å), while the distances of the others are all over 5.0 Å (Fig. S3(c)). Charge distributions on the closest I_2_ molecule are −0.141 and −0.177, respectively. The polarization of this I_2_ molecule, the attraction between this I_2_ and the aperture are both stronger than that of Cl_2_ or Br_2_. In the stable state, only one I_2_ is located on top of the aperture center, as shown in Fig. 3. This is because that the bond length of I_2_ is 2.863 Å, much longer than that of Cl_2_ (2.490 Å) and Br_2_ (2.510 Å), taking up space upon the aperture. The shortest distance between I_2_ and the aperture is 3.221 Å, which is in good agreement with the experimental value of 3.210 Å obtained from difference Fourier analysis^46^. Charge distribution on I close to the aperture is changed to −0.240 and the other is −0.250, which are much less than that of Br_2_. Thus, the aperture has little variation. Me-m 1, 3, 5 are rotated by a slightly −0.9° in average, orienting to I_2_; Me-m 2, 4, 6 are rotated by 8.1°, orienting to Z-axis. Correspondingly, *E*_T_ of I_2_ (−43.0 kJ mol^−1^, which is in good agreement with the previous research^47^) is the minimum of the three halogens. Therefore, the adsorption rate of I_2_ is the slowest in the initial adsorption stage of the experiment.

When the straight-line distance between I_2_ and the aperture shortens, the mIms gradually rotate from 35.1° to 0.64°, like a switch turned off. Only one state with an imaginary frequency of 391 *i* cm^−1^ which I_2_ is perpendicular to the aperture plane is found. As shown in Fig. 5(d), the distances between I_2_ molecule and mIm 1, 3, 5 are 3.956, 4.001 and 3.999 Å, respectively. Charge distributions on each I are −0.219 and −0.219, which has less steric electric effect than Br_2_ to the protons. When I_2_ molecules get inside, simulated result reveals that a maximum of 5 I_2_ molecules can stabilize in the cage, which like a critical feature that retains I_2_ in ZIF-8 until the framework collapses. According to the experimental data (Fig. S8, 0.97 g I_2_/g), each cage contains 5.2 I_2_ molecules, which is in good agreement with the previous research determined by thermogravimetric analysis (5.4 I_2_ in each cage)^48^. MD simulation shows that each I_2_ molecule locates close to the center of the aperture (Fig. S9) and the molecules only move around the localized locations.

**Figure 7:**
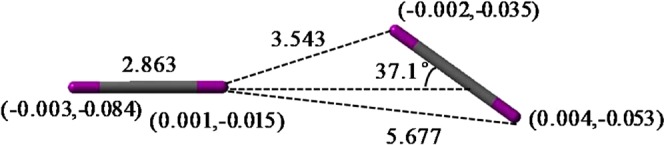
The typical model of intermolecular I_2_ − I_2_ interaction separated from the entire optimized structure. Bond distances are in Å and the charges are listed in parentheses (the former is not affected by the framework of cages, while the latter is affected).

In desorption process, possible halogen bond interactions are further investigated. Although the halogen bonds are weakened under the influence of framework of cages, there is an interaction between Br_2_ or I_2_ aggregated molecules. For example, charge distributions reveal the slight influence of framework on I_2_ molecules (Fig. S9). A model of I_2_ − I_2_ interaction including bond distances, bond angles and charge distributions separated from the entire structure is shown in Fig. 7. The shortest distance between two molecules is 3.543 Å and the bond angle is 37.1°, which are in consistent with the values in polyiodide and metal iodide−iodine systems^47^. Without the influence of framework, I_2_ would be polarized and there would be an attraction between the two molecules, the energy of which is 9.1 kJ mol^−1^. Charge distributions on one I_2_ molecule would be −0.003 and 0.001, the other are −0.002 and 0.004. In the cage, charges of I_2_ molecules are changed to −0.084 and −0.015, −0.035 and −0.053, respectively. The attraction energy is reduced to about 3.0 kJ mol^−1^ but not disappeared. Therefore, halogen bonds can be considered as another factor to the overall stabilization.

Based on the above analysis, it is quite possible that I_2_ in part of the upper layer of cages are regularly fixed and the diffusion rate is slowed down. That is why in the experiment, the amount of Br_2_ adsorbed increases slightly more than that of I_2_ in the initial 30 min. Also, the halogen bonds increase the overall stabilization. Thus in the desorption experiment (Fig. S8), the decreased amount of I_2_ (54%) is the vapor adsorbed on the surface of ZIF-8 film before. In addition, it has been proved by XRD and FTIR that interaction between I_2_ and mIms is the main influence factor of I_2_ fixation and after sorption, the cage does not reveal any significant changes^46,49,50^.

## Conclusions

In this work, the detailed attachment, diffusion and desorption processes of halogens in ZIF-8 have been explored by simulation on dynamics and thermodynamics. Deep interpretations including structure parameters, charge distributions and energy variations are discussed. The *E*_T_ of Cl_2_, Br_2_ and I_2_ are −55.2, −48.5 and −43.0 kJ mol^−1^, respectively. The framework of ZIF-8 is disrupted with the interaction of Cl_2_ and Zn on the surface and in the cage. Framework deformation on the surface of ZIF-8 can be caused by the attachment of Br_2_ but not I_2_. In diffusion, vapor molecules tend to vertically pass through the aperture of cages and a maximum of 7 Br_2_ or 5 I_2_ molecules can be accommodated with different states in each cage. In desorption process, the decrease of Br_2_ or I_2_ is the molecules in the state of non-regular thermal motion in the cages or adsorbed on the surface of ZIF-8 before. Next, more ZIFs materials served as adsorbents will be investigated by simulation and some regularities will be summarized. It also hopes to design more MOFs materials with better adsorption properties and performances based on the simulation method.

## Supplementary information

Supplementary Information.
