# Supplementary material for: Computational characterization of halogen vapor attachment, diffusion and desorption processes in zeolitic imidazolate framework-8
Source: Sci Rep. 2020 Feb 20;10:3010. doi: 10.1038/s41598-020-59871-x (PMC7033102; doi:10.1038/s41598-020-59871-x)
Supplement: Supplementary file 1 — Supplementary Information. [file 41598_2020_59871_MOESM1_ESM.docx]

**Computational characterization of halogen vapor attachment, diffusion and desorption processes in zeolitic imidazolate framework-8**

Dejie Li, Ying Han, Deqiang Li, Qi Kang, Dazhong Shen

Corresponding author.

E-mail address: wangyi3528@163.com dzshen@sdnu.edu.cn (D. Shen)

**Figure S1.** A three dimensional view of the relevant hexagonal window aperture selected.

**Figure S2.** The designed (D) and optimized (O) 8 different aperture structures based on the swing effect.

**Figure S3.** Snapshots of halogen molecules gathering together on the top of the aperture in the attachment process.

**Figure S4.** MD simulation of interaction between Cl_2_ molecule and C7 in the diffusion process.

**Figure S5.** Schematic drawing of experimental setups (not to scale) employed for equivalent circuit parameters (A) and absorption/desorption measurements (B).

**Figure S6.** Shifts of the resonant frequency of EL-QCM during the adsorption/desorption stages of Br_2_ vapor.

**Figure S7.** The bond distances of Br_2_ molecules in one cage of ZIF-8.

**Figure S8.** Shifts of the resonant frequency of EL-QCM during the adsorption and desorption stages of I_2_ vapor.

**Figure S9.** The schematic diagram of interaction between 5 I_2_ molecules and the cage of ZIF-8.

**Table S1.** Bond lengths (Å) in the most stable aperture structure

**Table S2.** Charge distributions (a.u.) in the most stable aperture structure

**Figure S1.** A three dimensional view of the relevant hexagonal window aperture selected. Zn, C and N atoms are shown in pink, gray and blue colors, respectively. H are omitted for clarity in the figure.


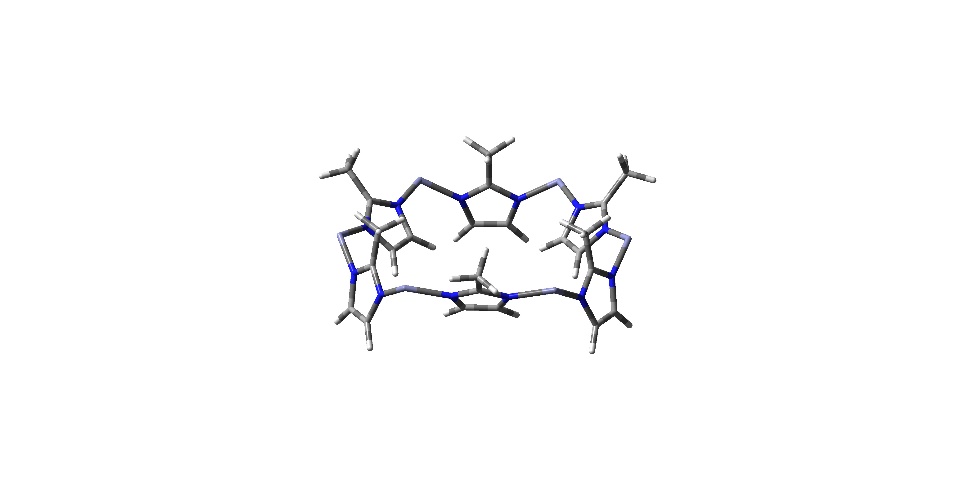

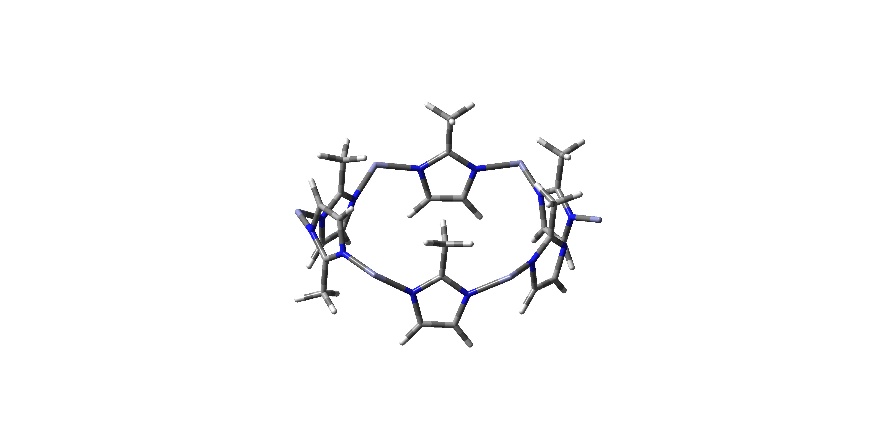

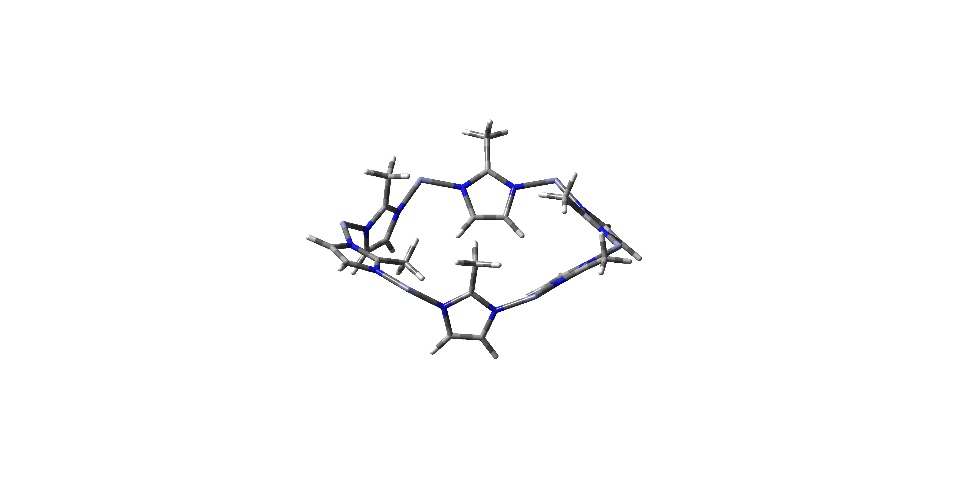


S-A (D) S-A (O) S-B (D) S-B (O)


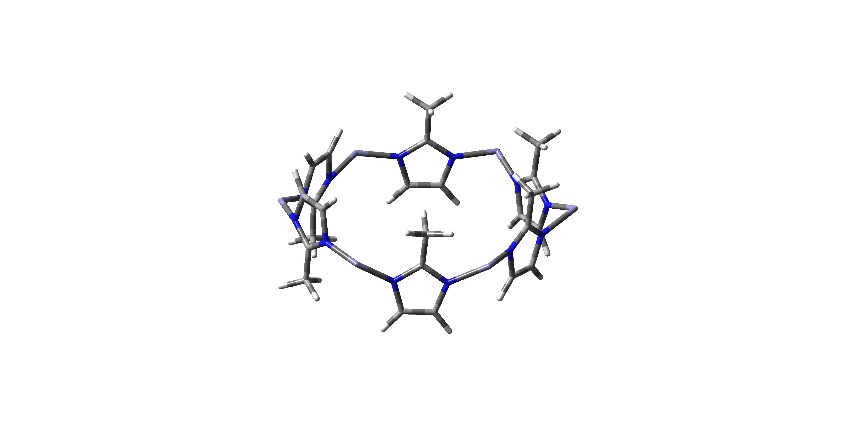

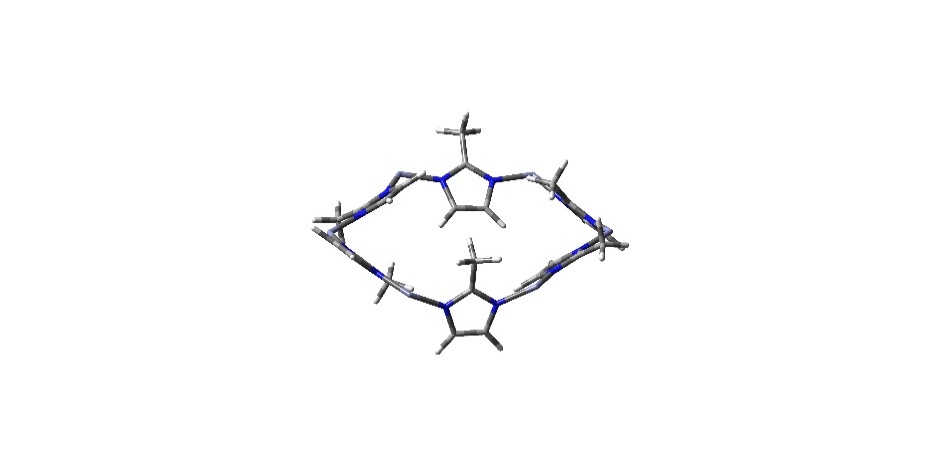

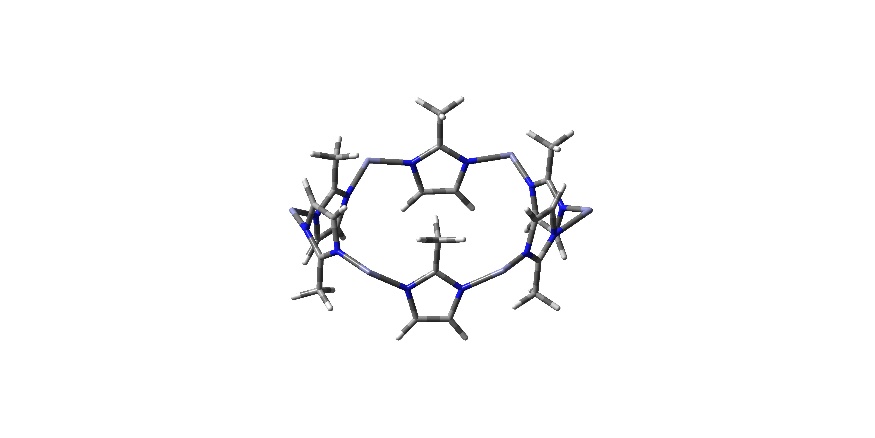

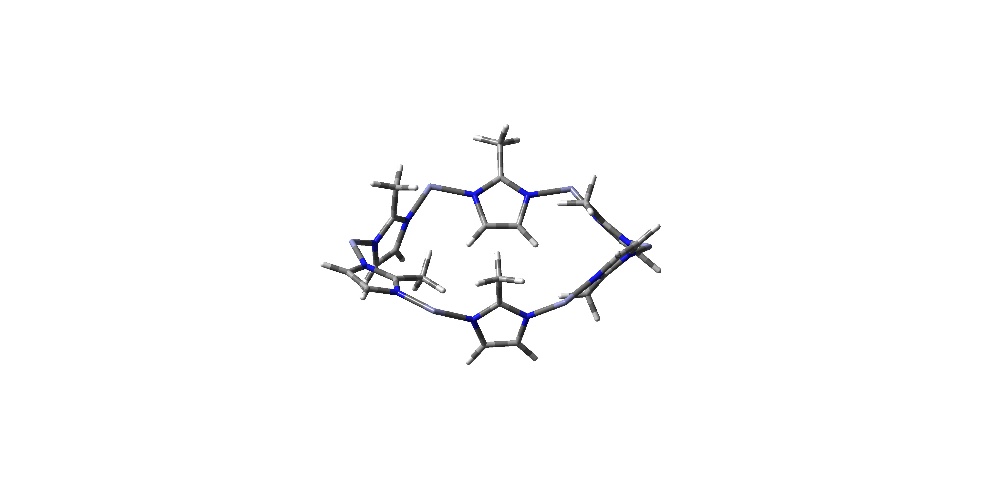


S-C (D) S-C (O) S-D (D) S-D (O)


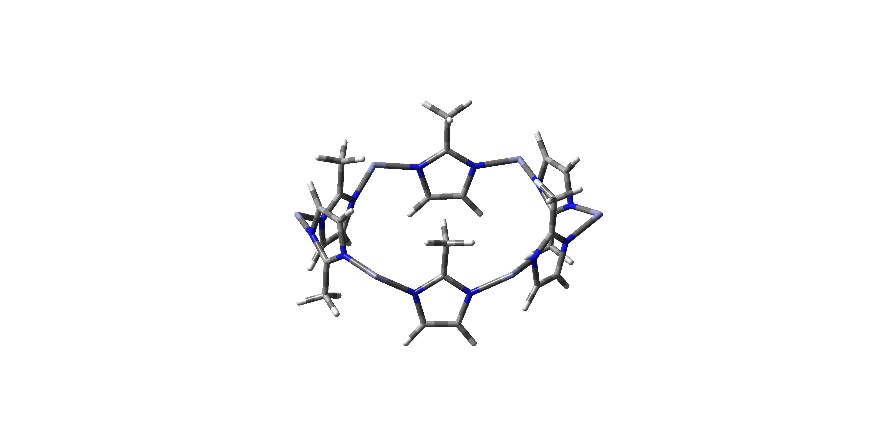

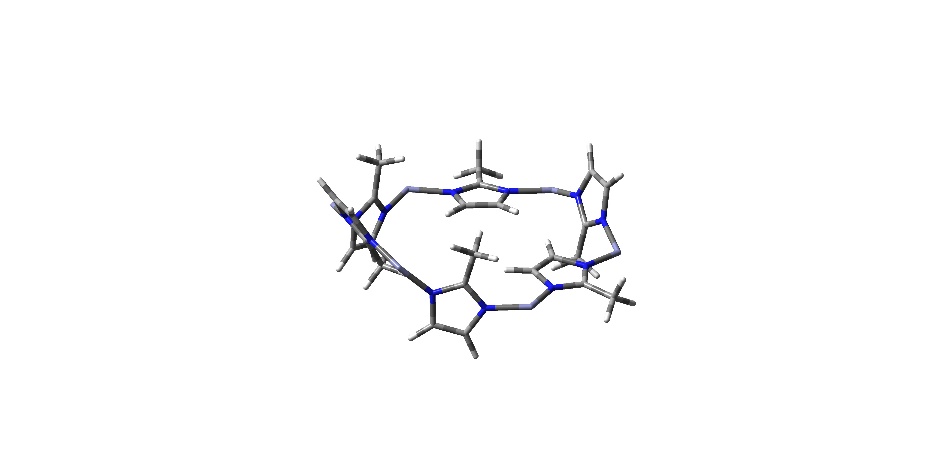

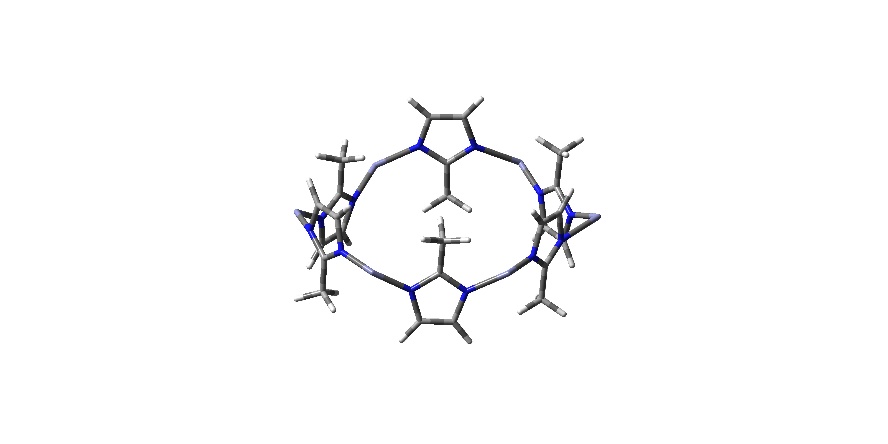

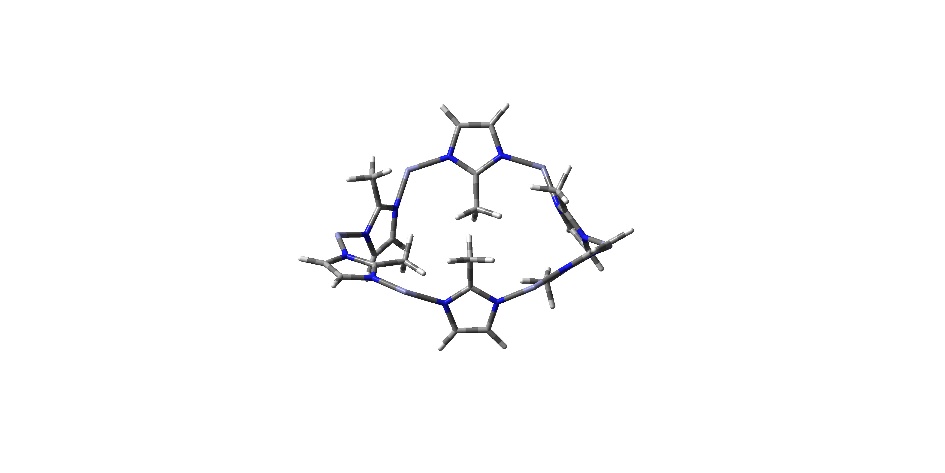


S-E (D) S-E (O) S-F (D) S-F (O)


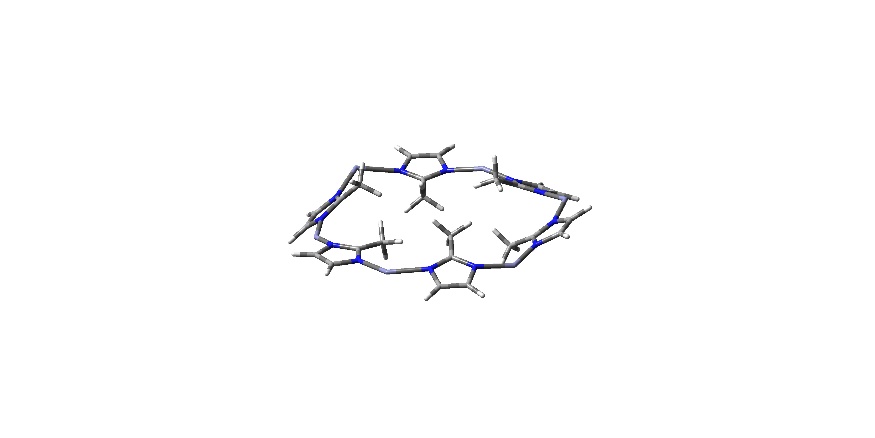

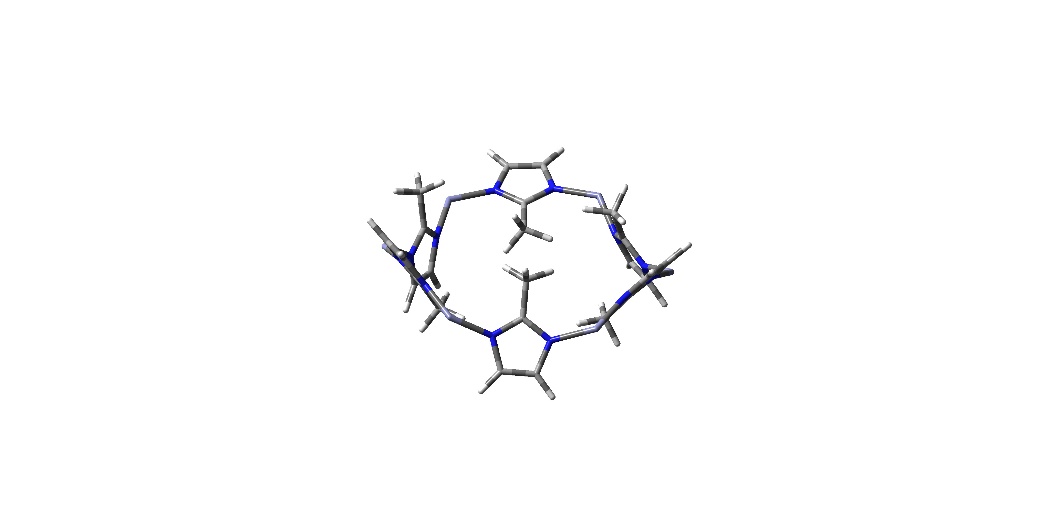

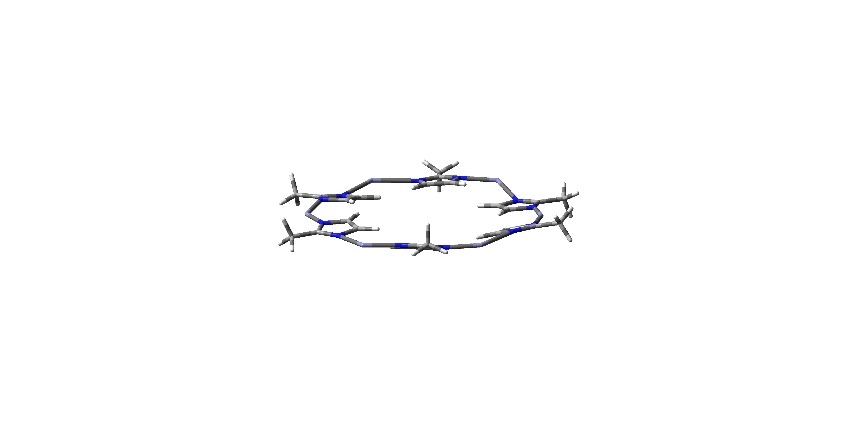

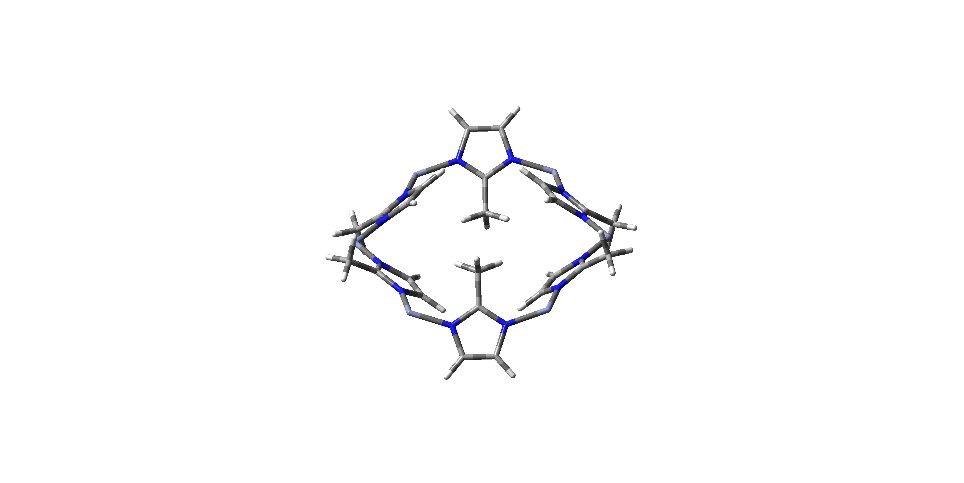


S-G (D) S-G (O) S-H (D) S-H (O)

**Figure S2.** The designed (D) and optimized (O) 8 different aperture structures based on the swing effect. Structures simulated are constituted with mIms groups originally, to show more clearly, other mIms groups are removed.

**Figure S3.** Snapshots of halogen molecules gathering together on the top of the aperture in the attachment process. Structures are got from MD simulation and further optimized by DFT. Charges are listed in parentheses: the former is not affected by the framework of cages, while the latter is affected. Bond distances are in Å.

**Figure S4.** MD simulation of interaction between Cl_2_ molecule and C7 in the diffusion process. The lattices around the cell are removed for clarity.

**Figure S5.** Schematic drawing of experimental setups (not to scale) employed for equivalent circuit parameters (**a**) and absorption/desorption measurements (**b**). (1) inlet for absorbate; (2) upper excitation electrode; (3) absorbate film; (4) QCM sensor; (5) magnetic mixer; (6) bottom excitation electrode; (7) thermostatic water bath.

**Figure S6.** Shifts of the resonant frequency of EL-QCM during the adsorption/desorption stages of Br_2_ vapor. The downward arrow indicates the addition of Br_2_ and the upward arrows indicate the start of desorption by vacuumizing operation. The frequency shifts were measured with the mIm and ZIF-8 films deposited, respectively.

**Figure S7.** The bond distances of Br_2_ molecules in one cage of ZIF-8. (**a**) 4 Br_2_ molecules in one cage, (**b**) 5 Br_2_ molecules in one cage, (**c**) 6 Br_2_ molecules in one cage, (**d**) 7 Br_2_ molecules in one cage. Bond distances are in Å.

**Figure S8.** Shifts of the resonant frequency of EL-QCM during the adsorption and desorption stages of I_2_ vapor. The downward arrow indicates the addition of I_2_ and the upward arrow indicates the start of desorption by vacuumizing operation.

**Figure S9.** The schematic diagram of interaction between 5 I_2_ molecules and the cage of ZIF-8. Charges are listed in parentheses: the former is not affected by the framework of cages, while the latter is affected. Bond distances are in Å.

**Table S1.** Bond lengths (Å) in the most stable aperture structure

| C9–C10 | C10–N11 | N11–C7 | C7–N8 | C7–C1 | N11–Zn12 |
| --- | --- | --- | --- | --- | --- |
| 1.382 | 1.404 | 1.369 | 1.369 | 1.501 | 2.078 |

**Table** **S2.** Charge distributions (a.u.) in the most stable aperture structure

| C9 | C10 | N11 | C7 | N8 | C1 | Zn12 | Zn13 |
| --- | --- | --- | --- | --- | --- | --- | --- |
| -0.071 | -0.070 | -0.813 | 0.456 | -0.811 | -0.691 | 0.873 | 0.867 |
